# Supplementary material for: GoldPolish-target: targeted long-read genome assembly polishing
Source: BMC Bioinformatics. 2025 Mar 7;26:78. doi: 10.1186/s12859-025-06091-7 (PMC11887200; doi:10.1186/s12859-025-06091-7)
Supplement: Supplementary file 1 — Additional file 1. [file 12859_2025_6091_MOESM1_ESM.docx]

**Supplementary Figures**

**
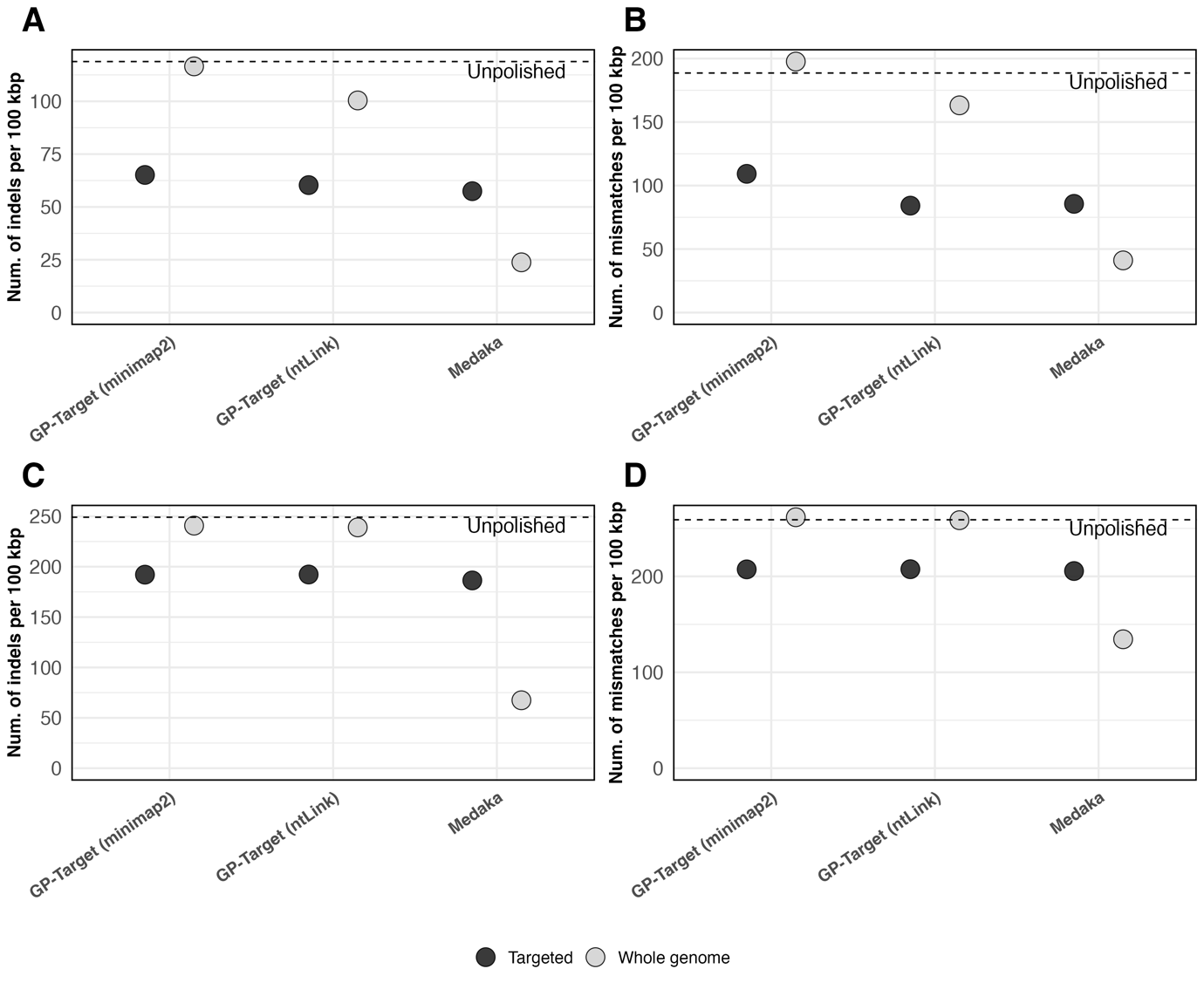
**

**Supplementary Figure 1. Indels and base mismatches before and after polishing GoldRush assemblies with GP-Target and Medaka.** The draft assemblies were polished with GP-Target with minimap2 mapping, denoted as GP-Target (minimap2), GP-Target with ntLink mapping, denoted as GP-Target (ntLink), and Medaka. The numbers on the y-axes, determined by QUAST, represent the A) number of indels per 100 kbp and B) the number of mismatches per 100 kbp before and after polishing the *D. melanogaster* assembly and C) the number of indels per 100 kbp and D) the number of mismatches per 100 kbp before and after polishing the *H. sapiens* draft GoldRush assembly. Each tool was used to polish the assembly in a targeted manner and in a non-targeted (whole genome) manner.


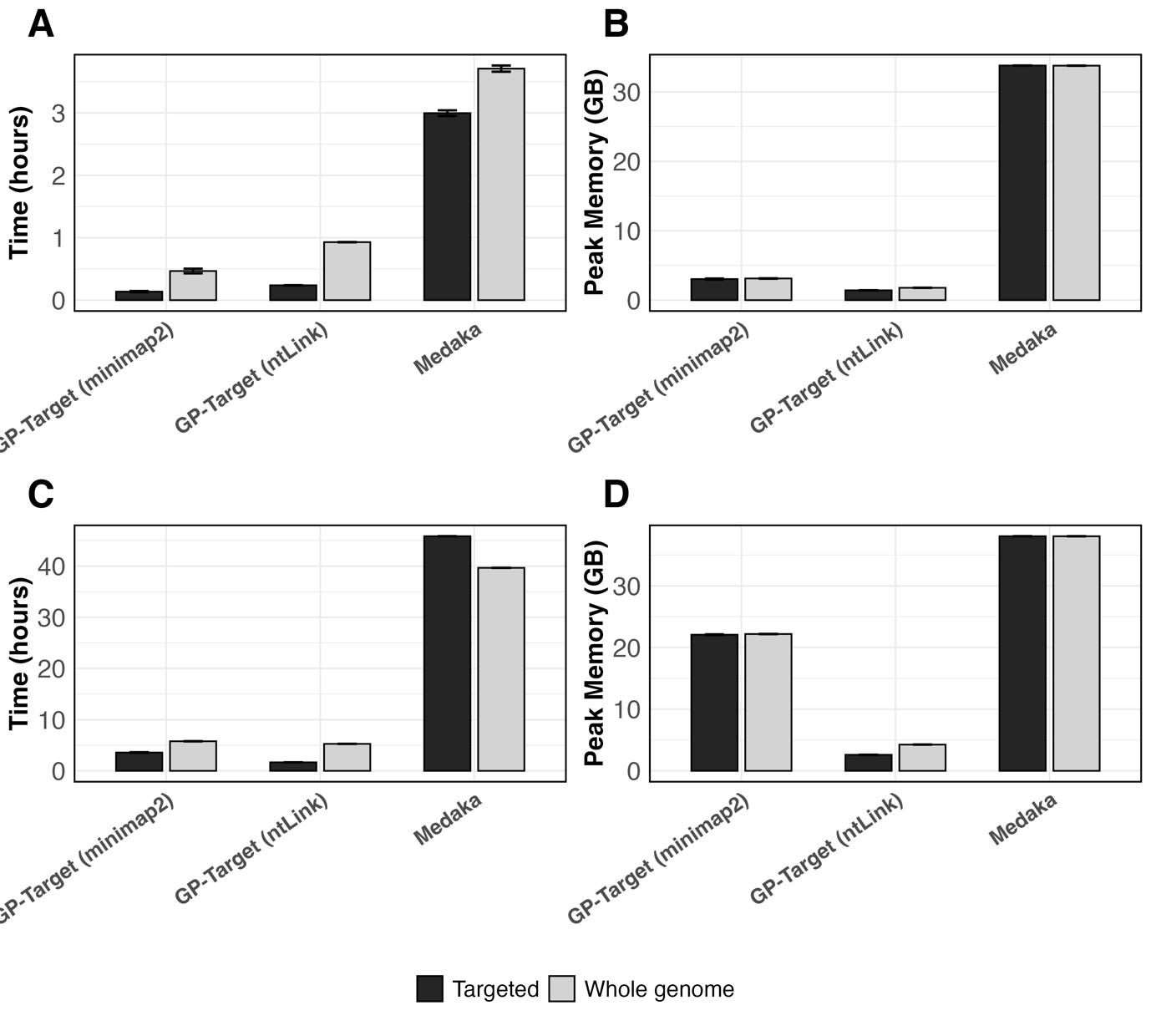


**Supplementary Figure 2. Compute resource usage of targeted polishing with GP-Target and Medaka**. *D.* *melanogaster* draft assembly polishing **A)** wall-clock time (in hours) and **B)** peak memory (RAM, in gigabytes) and *H.* sapiens draft assembly polishing **C)** wall-clock time (in hours) and **D)** peak memory (RAM, in gigabytes) associated with GP-Target and Medaka. GP-Target with minimap2 alignment is denoted as GP-Target (minimap2) and GP-Target with ntLink mapping is denoted as GP-Target (ntLink). Polishing only the target regions is denoted as Targeted and polishing the whole genome is denoted as Whole Genome. 48 threads were specified for all benchmarking runs.


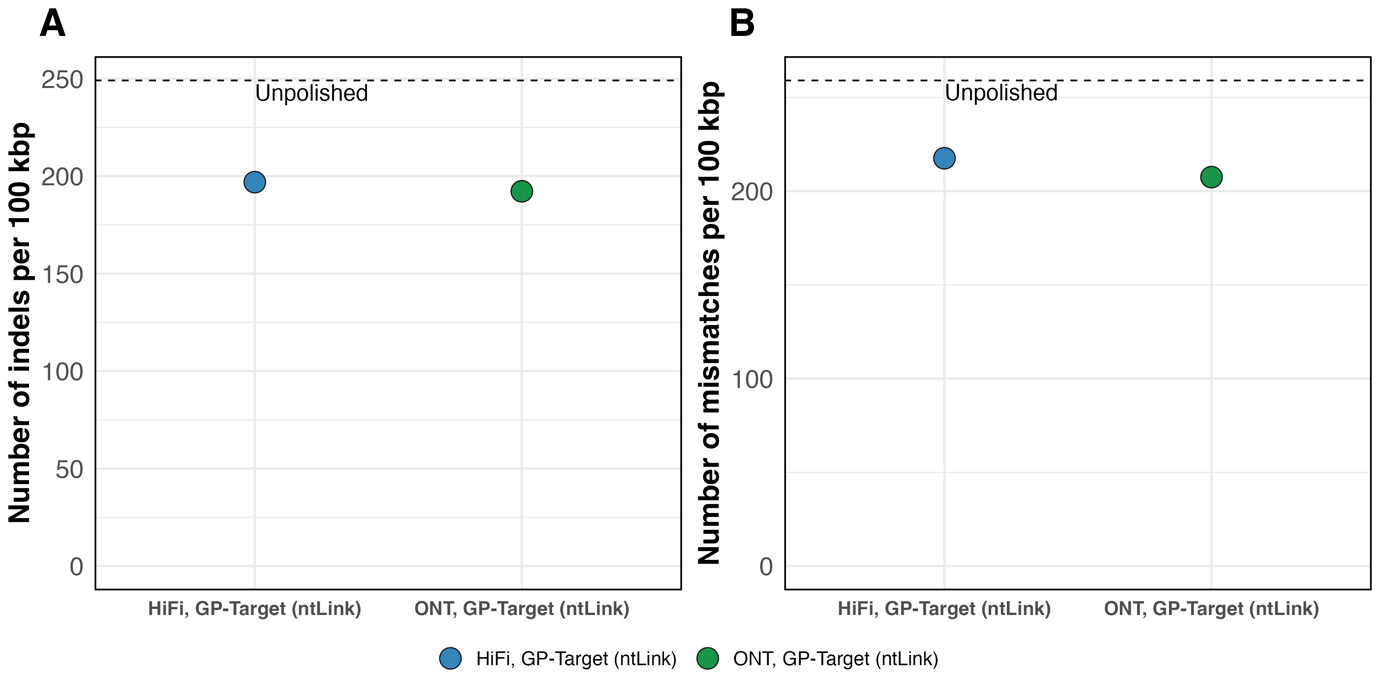


**Supplementary Figure 3. Indels and base mismatches before and after polishing GoldRush assemblies with ONT long reads and PacBio HiFi reads.** The draft assemblies were polished with GP-Target with ntLink mapping, denoted as GP-Target (ntLink). The numbers on the y-axes, determined by QUAST, represent the A) number of indels per 100 kbp, B) the number of mismatches per 100 kbp before and after polishing the *H. sapiens* draft GoldRush assembly.

**Supplementary Tables**

**Supplementary Table 1. ONT long-read sequencing reads used for genome assembly assessments**. The N50 length and associated base error rate for each data set were assessed with ABySS (1) and NanoSim (2), respectively.

| **Species** | **Fold Coverage** | **N50 Length (bp)** | | **Accession(s)/ Source** | **Basecaller** | **Flowcell** | **Estimated Error Rate (%)** |
| --- | --- | --- | --- | --- | --- | --- | --- |
| *D. melanogaster* | 154 | 19,281 | SRR22822929 | | Guppy v6 | R10.4.1 | 3 |
| *H. sapiens* | 67 | 30,348 | s3://ont-open-data/gm24385_2020.11/analysis/r9.4.1/20201026_1644_2-E5-H5_PAG07162_d7f262d5/guppy_v4.0.11_r9.4.1_hac_prom/align_unfiltered/chr1/guppy_v5.0.6_r9.4.1_sup_prom/ | | Guppy v5 | R4.9.1 | 4 |

**Supplementary Table 2. Reference genomes used for QUAST assembly base quality evaluation**. Reference genomes for *D. melanogaster* and *H. sapiens* were sourced from the NCBI Reference Sequence Database and the Genome Reference Consortium (3), respectively.

| **Species** | **Reference genome build** | **Accession** |
| --- | --- | --- |
| *D. melanogaster* | Release 6 | NC_004354 |
| *H. sapiens* | GRCh38 | GCA_000001405.15 |

**Supplementary Table 3. Short read datasets used for Merqury assembly base quality evaluation**. The datasets were obtained from the Sequence Read Archive (SRA).

| **Species** | **SRA Accession** |
| --- | --- |
| *D. melanogaster* | SRR11460799 |
| *H. sapiens* | SRR11321732 |

**Supplementary Table 4.** PacBio long reads for polishing the *H. sapiens* NA24835 cell line draft assembly. The dataset was obtained from the Sequence Read Archive (SRA).

| **Data** | **SRA Accession** | **Chemistry** |
| --- | --- | --- |
| CCS HiFi long reads | SRX5327410 | Sequel Sequencing Kit 3.0 chemistry |

**Supplementary Table 5. BUSCO statistics for the *D. melanogaster* assembly before and after polishing.** BUSCO was run with diptera_odb10 lineage and 3,285 BUSCO groups were searched (4).

| **Assembly** | **Complete BUSCOs (C)** | **Complete and single-copy BUSCOs (S)** | **Complete and duplicated BUSCOs (D)** | **Fragmented BUSCOs (F)** | **Missing BUSCOs (M)** |
| --- | --- | --- | --- | --- | --- |
| Unpolished | 3,213 (97.8%) | 3,104 | 109 | 28 | 44 |
| Medaka | 3,221 (98.0%) | 3,006 | 215 | 23 | 41 |
| GP-Target (minimap2) | 3,219 (98.0%) | 2,992 | 227 | 24 | 42 |
| GP-Target (ntLink) | 3,219 (98.0%) | 2,998 | 221 | 25 | 41 |

**Supplementary Table 6.** **BUSCO statistics for the *H. sapiens* NA24385 cell line draft assembly before and after polishing.** BUSCO was run with primates_odb10 lineage and 13,780 BUSCO groups were searched (4).

| Tool | Complete BUSCOs (C) | Complete and single-copy BUSCOs (S) | Complete and duplicated BUSCOs (D) | Fragmented BUSCOs (F) | Missing BUSCOs (M) |
| --- | --- | --- | --- | --- | --- |
| Unpolished | 12,158 (88.2%) | 11,931 | 227 | 514 | 1,108 |
| Medaka | 12,290 (89.2%) | 11,879 | 411 | 506 | 984 |
| GP-Target (minimap2) | 12,264 (89.0%) | 11,850 | 414 | 508 | 1,008 |
| GP-Target (ntLink) | 12,274 (89.1%) | 11,868 | 406 | 510 | 996 |

**Supplementary Table 7. Correctness statistics of *D. melanogaster* genome assembly before and after polishing.** All statistics were generated using QUAST (5).

| Tool | Polishing regions | Number of mismatches per 100 kbp | Number of indels per 100 kbp |
| --- | --- | --- | --- |
| Unpolished | N/A | 188.61 | 118.81 |
| Medaka | Targeted | 85.59 | 57.45 |
| Medaka | Whole genome | 41.07 | 23.78 |
| GP-Target (minimap2) | Targeted | 109.22 | 65.14 |
| GP-Target (minimap2) | Whole genome | 197.63 | 116.54 |
| GP-Target (ntLink) | Targeted | 84.17 | 60.33 |
| GP-Target (ntLink) | Whole genome | 163.17 | 100.41 |

**Supplementary Table 8. Correctness statistics of *H. sapiens* cell line *H. sapiens* NA24385 cell line genome assembly before and after polishing.** All statistics were generated using QUAST (5).

| Tool | Polishing regions | Polishing reads | Number of mismatches per 100 kbp | Number of indels per 100 kbp |
| --- | --- | --- | --- | --- |
| Unpolished | N/A | N/A | 259.01 | 249.1 |
| Medaka | Targeted | ONT | 205.56 | 186.37 |
| Medaka | Whole genome | ONT | 134.42 | 67.43 |
| GP-Target (minimap2) | Targeted | ONT | 207.3 | 192.1 |
| GP-Target (minimap2) | Whole genome | ONT | 261.65 | 240.77 |
| GP-Target (ntLink) | Targeted | ONT | 207.46 | 192.25 |
| GP-Target (ntLink) | Whole genome | ONT | 258.56 | 239.04 |
| GP-Target (ntLink) | Targeted | PacBio | 217.52 | 196.93 |

**Supplementary Table 9. Percentage of genome polished, and compute resource usage associated with polishing the *D. melanogaster* draft assembly.** All benchmarking runs were run with 48 threads specified in the command. The reported values for wall-clock time and peak memory usage are the mathematical mean of triplicate runs.

| Tool | Percentage of genome polished | Mean wall-clock time (hours) | Mean peak memory (RAM, in gigabytes) |
| --- | --- | --- | --- |
| Medaka | 15.43% | 3.00 | 33.82 |
| Medaka | 100% | 3.71 | 33.80 |
| GP-Target (minimap2) | 15.43% | 0.14 | 3.04 |
| GP-Target (minimap2) | 100% | 0.47 | 3.12 |
| GP-Target (ntLink) | 15.43% | 0.24 | 1.41 |
| GP-Target (ntLink) | 100% | 0.93 | 1.78 |

**Supplementary Table 10. Proportion of genome polished, and compute resource usage associated with polishing the *H. sapiens* NA24385 cell line draft assembly.** All benchmarking runs were run with 48 threads specified in the command. The reported values for wall-clock time and peak memory usage are the mathematical mean of triplicate runs.

| Tool | Percentage of genome polished | Mean wall-clock time (hours) | Mean peak memory (RAM, in gigabytes) |
| --- | --- | --- | --- |
| Medaka | 7.23% | 45.84 | 38.03 |
| Medaka | 100% | 39.66 | 38.03 |
| GP-Target (minimap2) | 7.23% | 3.58 | 22.07 |
| GP-Target (minimap2) | 100% | 5.78 | 22.18 |
| GP-Target (ntLink) | 7.23% | 1.66 | 2.60 |
| GP-Target (ntLink) | 100% | 5.27 | 4.25 |

**References**

1. Jackman SD, Vandervalk BP, Mohamadi H, Chu J, Yeo S, Hammond SA, et al. ABySS 2.0: resource-efficient assembly of large genomes using a Bloom filter. Genome Res. 2017 May;27(5):768–77.

2. Yang C, Chu J, Warren RL, Birol I. NanoSim: nanopore sequence read simulator based on statistical characterization. GigaScience [Internet]. 2017 Apr 1 [cited 2023 Dec 10];6(4). Available from: https://academic.oup.com/gigascience/article/doi/10.1093/gigascience/gix010/3051934

3. Cunningham F, Allen JE, Allen J, Alvarez-Jarreta J, Amode MR, Armean IM, et al. Ensembl 2022. Nucleic Acids Res. 2022 Jan 7;50(D1):D988–95.

4. Manni M, Berkeley MR, Seppey M, Zdobnov EM. BUSCO: Assessing Genomic Data Quality and Beyond. Curr Protoc. 2021 Dec;1(12):e323.

5. Mikheenko A, Prjibelski A, Saveliev V, Antipov D, Gurevich A. Versatile genome assembly evaluation with QUAST-LG. Bioinformatics. 2018 Jul 1;34(13):i142–50.
